# Immunization during pregnancy: do healthcare workers recommend vaccination against influenza?

**DOI:** 10.3389/fpubh.2023.1171142

**Published:** 2023-06-02

**Authors:** Francesca Licata, Concetta Paola Pelullo, Giorgia Della Polla, Emma Antonia Citrino, Aida Bianco

**Affiliations:** ^1^Department of Health Sciences, School of Medicine, University of Catanzaro “Magna Graecia”, Catanzaro, Italy; ^2^Department of Movement Sciences and Wellbeing, University of Naples “Parthenope”, Naples, Italy; ^3^Health Direction, Teaching Hospital, University of Campania “Luigi Vanvitelli”, Naples, Italy

**Keywords:** COVID-19 vaccine, healthcare workers, immunization, influenza vaccine, pregnancy, vaccine preventable diseases

## Abstract

**Background:**

A variety of circumstances can influence how widely vaccination during pregnancy is accepted. Healthcare workers (HCWs) are often seen as the main resource for recommending vaccination. The purpose of the current study was to determine whether Italian HCWs advise and recommend pregnant people to receive the influenza vaccinations, as well as what knowledge and attitudes affect their practices. A secondary aim of the study was to evaluate HCWs’ knowledge and attitudes towards COVID-19 vaccination.

**Methods:**

This cross-sectional study, took place between August 2021 and June 2022 in a randomly selected sample of HCWs in three Italian regions. The target population comprised obstetricians-gynecologists, midwives and primary care physicians, who provide medical care to pregnant people. The questionnaire consisted of 19 items divided into 5 parts gathered information about the participants’ sociodemographic and professional characteristics, general knowledge about vaccinations during pregnancy, and vaccine-preventable diseases (VPDs), attitudes and practices towards immunization, and strategies to potentially increase vaccination uptake during pregnancy.

**Results:**

Among the participants, 78.3% knew that pregnant people are at increased risk of severe complications from influenza, 57.8% that the influenza vaccine is not provided only in the 2nd/3rd trimester of pregnancy and 60% that pregnancy is a risk factor for severe COVID-19 infection. Of the enrolled HCWs, 10.8% believed that the potential risks of vaccines administered during pregnancy are greater than the benefits. An even higher proportion of the participants was unsure (24.3%) or did not deem (15.9%) that vaccinating against influenza during pregnancy reduces the risk of preterm birth and abortion. Moreover, 11.8% of the sample did not believe or was uncertain that COVID-19 vaccine must be offered to all pregnant people. Among HCWs, 71.8% advised women about influenza vaccination during pregnancy, and 68.8% recommended getting vaccinated against influenza during pregnancy. Results showed that good knowledge and positive attitudes were the strongest factors positively associated with advising women about influenza vaccination during pregnancy.

**Conclusion:**

The gathered data showed that a sizable portion of the HCWs lacks up-to-date knowledge, underestimates the risks of contracting a VPD, and overestimates the risks of vaccine side effects during pregnancy. The findings shed light on such attributes useful to promote adherence to evidence-based recommendations among HCWs.

## 1. Introduction

Infections during pregnancy are widely recognized to raise the risk of serious illnesses for women, have negative effects on the development of the fetus, and increase mortality rates (1).

Maternal immunization has received substantial and growing attention in recent years, and observational studies have shown that doing so is a safe and highly valuable public health measure that benefits the mother, the developing fetus, and the young newborn (2, 3). Therefore, vaccination programmes for expectant mothers have been implemented in several countries, including Italy, to protect newborns through passive immunity that is passed on naturally (4). The Diphtheria, Tetanus, Pertussis (Tdap) vaccine (recommended for administration during the 27th through the 36th week of pregnancy) and influenza vaccine (recommended for administration at any time) are both included in the Italian Ministry of Health’s National Immunization Plan (2017–2019) (5) adopted by the Italian Ministry of Health. In addition to this, the 2020–2021 influenza season saw increased importance for influenza vaccination due to the unique pandemic coronavirus disease 2019 (COVID–19), which was brought on by the severe acute respiratory syndrome coronavirus 2 (SARS-CoV-2). Both respiratory viral infections have the high-risk and similar symptoms and are extremely dangerous for expectant mothers. Consequently, influenza and COVID-19 vaccines are recommended during each pregnancy (6).

The Italian Ministry of Health defined the minimum level of vaccination coverage of 75% with an optimal target of 95% in accordance with recommendations from the World Health Organization (WHO) (7) and the European Centre for Disease Prevention and Control (ECDC) (5). In Italy, in order to achieve the aforementioned goals, several influenza vaccines received Italian approval for the 2020–2021 influenza season (8). Among these, pregnant people should get either the inactivated influenza vaccination (IIV) or the recombinant influenza vaccine (RIV) throughout flu season (9). It is difficult to pinpoint the reasons for the dramatic reduction in the uptake of influenza vaccination that happened between 2005–2006 and 2014–2015 in Italy. In reality, a variety of circumstances can influence how widely vaccination is accepted (10). Some potential factors for missed influenza vaccination have been suggested by recent investigations (11–13). These include a lack of understanding of the advantages of vaccination, inconsistent medical advice, and questions regarding the efficacy of vaccines (14). Healthcare workers (HCWs) are often seen as the main resource for promoting and recommending vaccination, especially to high-risk population (1, 14). Similarly, at the beginning of 2021 international (15, 16) and national guidelines (17) recommended HCWs to provide information and counseling about the vaccine to pregnant people at high risk for severe COVID-19. Afterwards, due to large evidence supporting safety, immunogenicity and efficacy of mRNA COVID-19 vaccines during pregnancy, international (18–20) and national health authorities (21) recommended the vaccine to all women at any point in pregnancy, as well as booster doses when it is time to get one. Therefore, the primary aim of the current study was to determine whether Italian HCWs advise and recommend pregnant people to receive the influenza vaccinations, as well as what attitudes affect their practices. A secondary aim was to explore HCWs’ knowledge and attitudes towards COVID-19 vaccination during pregnancy. Moreover, we also made the decision to evaluate their level of expertise on the subject, and how valuable they think their role is in implementing vaccination uptake during pregnancy to better identify strategies to increase vaccine uptake.

## 2. Materials and methods

### 2.1. Study design and setting

This study was designed as a cross-sectional survey, taking place between August 2021 and June 2022 in a sample of HCWs from three Southern Italian Regions (i.e., Calabria, Campania, and Sicily).

### 2.2. Study population and data collection

The target population comprised obstetricians-gynaecologists (OB/GYNs), midwives, and primary care physicians (PCPs), who provide medical care to women during pregnancy. The exclusion criterion was not having a good command of the Italian language. The survey was conducted in 2 steps. In the first one, a random sample of five facilities that supply prenatal care were randomly selected for each region. Following that, a letter was sent to the management staff to explain the study’s purpose and to obtain written permission to conduct the survey in the facility. In the second step, e-mails containing a URL directing to the homepage of the online survey were sent to 50 randomly selected OB/GYNs and midwives among those practicing at each selected facility. Similarly, 50 PCPs were randomly recruited from the publicly available frames among those provided health care within the Health Service of each selected region. The link to the questionnaire was personal; it contained a unique serial number but no personal identifiers. Nonrespondents received a reminder after 2, 4 and 8 weeks. In an attempt to maximise the response rate, data were also collected through a self-administered paper questionnaire distributed by trained medical personnel or through a QR code that immediately directed to the electronic survey. All participants were informed about the survey’s background, objectives, and privacy policies. Participants learned about the anonymity and confidentiality of collected data through the informed consent document. Recruited individuals were aware that they could withdraw from the study at any time and that there was signposting to support services if participants felt they needed it. Before filling out the questionnaire, the investigators obtained a written informed consent. For participants that filled out the online questionnaire, on the first page of the survey, there was an informed consent form at the end of which participants could give their agreement to join the study. Participants were encouraged to print a copy or save a pdf of the informed consent for their records. Respondents received no compensation or incentives for taking part in this study.

### 2.3. Sample size

The sample size was calculated using a 5% margin of error, a 95% confidence level, and a hypothetical 50% response distribution based on the prevalence of recommending influenza vaccine to pregnant people among HCWs (22–25). Based on these assumptions, a sample of at least 380 HCWs was required. By anticipating a low response rate (26–30), the total sample size was inflated to 700 healthcare workers.

### 2.4. Questionnaire design

The questionnaire was designed following an extensive review of the literature (25, 28, 29, 31, 32), and it was pretested on a sample of 20 eligible HCWs not included in the final sample. Minor refinements to improve the flow and clarity of the tool were made. The final questionnaire had 19 items divided into 5 sections. It took about 10 min to finish all of the items. The first section of the questionnaire gathered information about the participants’ sociodemographic and professional characteristics (4 items, closed-ended items with multiple answers and open-ended items), such as age, gender, position, and the total number of years in practice. The second section (4 items with multiple-choice answers “true, false, do not know”) looked into general knowledge about vaccinations against influenza and COVID-19 during pregnancy, as well as about those vaccine-preventable diseases (VPDs). The third section (5 items on a 5-point Likert scale ranging from “strongly disagree” to “strongly agree”) assessed attitudes towards immunization during pregnancy. The fourth section (4 items with multiple answers and open options) investigated HCWs’ immunization practices (i.e., advising and recommending pregnant people to get vaccinated against influenza) and strategies and interventions to potentially increase vaccination uptake during pregnancy. The final section (2 items, closed-ended with multiple answers and open options) examined information sources, satisfaction with these sources, and the need for additional information about recommended vaccination during pregnancy. Ethical approval of the study was granted by the Calabria Centre Local Human Research Ethics Committee (ID No. 275/2021/07/15).

### 2.5. Statistical analysis

All collected variables were obtained by means and standard deviations (SD) when normally distributed. Medians and interquartile ranges (IQR) were calculated in cases of deviations from normality. Categorical variables were expressed in percentages. The knowledge score about vaccinations and VPDs during pregnancy was calculated by assigning one point for each right response and summing the scores for each statement (range 0–4). Similarly, an overall attitude was calculated by assigning a value from zero to the least positive response to four to the most positive one and summing the values of each statement (range 0–20). Two logistic regression models were developed to explore the role of potential predictors of HCWs’ practices regarding immunization during pregnancy: having advised women about influenza immunization during pregnancy (Model 1) and having recommended influenza immunization during pregnancy (Model 2). The following selected independent variables were included in the models: gender (male = 0; female = 1), profession (OB/GYN = 0; PCP = 1; midwife = 2), number of years in practice (continuous), knowledge score about vaccinations and VPDs during pregnancy (continuous), attitude score about immunization during pregnancy (continuous). In Model 2, the variable having advised pregnant people about influenza vaccination (no = 0; yes = 1) was also included. The Hosmer and Lemeshow test assessed the goodness of fit of the logistic model and visual investigation of the lowess curve fitting linear predictor (log-odds) values by Pearson Standardized residuals. Moreover, since OB/GYNs, PCPs and midwives are not homogenous in terms of medical education and training, further logistic regression analyses were performed to explore the potential predictors of the outcomes of interest among those different professional groups. The statistical significance level was fixed at a *p*-value <0.05. The adjusted odds ratio (OR) and 95% confidence interval (CI) were calculated. Statistical analysis was developed using the STATA software program, version 17 (33).

## 3. Results

### 3.1. Participants’ demographics

All selected facilities in the Calabria and Sicily regions agreed to participate in the study, whereas four out of five in the Campania region did. Out of 700 HCWs approached, 415 HCWs (59.3%) completed the questionnaire. In particular, of the 550 OB/GYNs and midwives who were approached, 386 (204 from Campania, 104 from Calabria and 51 from Sicily) agreed to participate, for a response rate of 70.2%. Of the 150 PCPs invited to participate in the study, 31 were not included because of incorrect e-mail addresses, and a total of 56 (36 from Calabria and 20 from Sicily) answered the questionnaire, giving a response rate of 47.1%. More than half (60.5%) of the participants were female, with an average age of 42.8 years (±11.3 SD). Almost two-thirds (64.8%) were OB/GYNs, 21.7% midwives and 13.5% PCPs, and the average number of years spent in practice was 14 (±11.5 SD).

### 3.2. HCWs’ knowledge related to vaccinations and VPDs during pregnancy

The overall median knowledge score was 3 (IQR 2–4) and just 28% of the sample correctly answered all 4 statements. Among the participants, 88.7% knew that the influenza vaccine administered during pregnancy protects both the woman and the newborn, 78.3% that pregnant people are at increased risk of severe complications from influenza and 57.8% that the influenza vaccine is not provided only in the 2nd/3rd trimester of pregnancy. Lastly, 60% of enrolled HCWs acknowledged that pregnancy is a risk factor for severe COVID-19 infection.

### 3.3. HCWs’ attitudes towards immunization during pregnancy

The median attitude score was 17 (IQR 15–18) and only 6.8% of the sample reached the maximum positive score of 20. Table 1 displays the responses to the statements about HCWs’ attitudes towards immunization during pregnancy. Of the enrolled HCWs, 10.8% believed that the potential risks of vaccines administered during pregnancy are greater than the benefits. An even higher proportion of the participants was unsure (24.3%) or did not deem (15.9%) that vaccinating against influenza during pregnancy reduces the risk of preterm birth and abortion. As regards the COVID-19 vaccine, 11.8% of the sample did not believe or was uncertain that it must be offered to all pregnant people (i.e., healthy and at the highest risk of getting very sick).

**Table 1:** HCWs’ attitudes towards immunization during pregnancy.

Statements (415 respondents)	Strongly disagree/ Disagree		Uncertain		Strongly agree/ Agree	
	*N*	%	*N*	%	*N*	%
Improving adherence to vaccinations during pregnancy is an effective strategy to prevent infectious diseases	8	1.9	8	1.9	**399**	**96.2**
Providing detailed information about effectiveness and safety of vaccinations is a useful strategy to improve vaccine uptake during pregnancy	2	0.5	13	3.1	**400**	**96.4**
The potential risks of vaccinations administered during pregnancy are greater than the benefits	**354**	**85.3**	16	3.9	45	10.8
Vaccinating pregnant people against influenza reduces the risk of preterm birth and abortion	66	15.9	101	24.3	**248**	**59.8**
COVID-19 vaccine must be offered to all pregnant people	19	4.6	30	7.2	**366**	**88.2**

Number and percentages referring to positive attitudes are in bold.

### 3.4. HCWs’ practices regarding immunization during pregnancy

Seven out of ten (71.8%) HCWs advised women about influenza vaccination during pregnancy. Among those who never, rarely, or sometimes do it, 44.4% were OB/GYNs, 42.7% midwives and 12.8% PCPs. The results of the regression analysis showed that good knowledge and positive attitudes were the strongest factors positively associated with advising women about influenza vaccination during pregnancy. Indeed, a 63% increase in the odds of having advised women about influenza immunization during pregnancy was shown for a one-unit increase in the knowledge score (OR: 1.63; 95% CI: 1.26–2.12). Similarly, a one-point increase in the attitude score led to a 20% increase in the odds of advising about influenza vaccination during pregnancy (OR: 1.20; 95% CI: 1.09–1.33). Furthermore, the odds of having advised pregnant people about influenza vaccination increased with every year in practice (OR: 1.02; 95% CI: 1.01–1.04), and it was less likely in midwives (OR: 0.29; 95% CI: 0.17–0.54) compared with OB/GYNs. The OB/GYNs’ advise about influenza immunization correlated positively and significantly with higher odds of attitude score (OR: 1.25; 95% CI: 1.09–1.44) and with the number of years in practices (OR: 1.04; 95% CI: 1.01–1.08). As far as it concerns both midwives and PCPs, the odds of having advised about influenza immunization was significantly higher among those with better knowledge score (OR: 2.04; 95% CI: 1.28–3.27) (Table data in the Supplementary material).

Among the sampled HCWs, 68.8% recommended getting vaccinated against influenza during pregnancy and, among those who never, rarely or sometimes recommended vaccination, 58.1% were OB/GYNs, 33.3% midwives and 8.5% PCPs. Among those who recommended the influenza vaccination, the vast majority (95.1%) recommended it to all pregnant people, 4.9% to high-risk women and 1.8% to women with chronic diseases, alone. The strongest predictor of this practice was having advised about vaccination against influenza during pregnancy (OR: 5.65; 95% CI: 3.35–9.54) (Model 2 in Table 2). Moreover, a one-unit increase in attitude score resulted in a 16% increase in the odds of having recommended influenza vaccination during pregnancy (OR: 1.16; 95% CI: 1.05–1.29). Moreover, the odds of having recommended vaccination against influenza to pregnant people were 2.45 times higher among PCPs (OR: 2.45; 95% CI: 1.07–5.59) compared with OB/GYNs. Subgroups analyses showed that having advised pregnant people about influenza immunization resulted in an increase in the odds of having recommended influenza immunization among PCPs (OR: 21.47; 95% CI: 2.91–158.39), as well as among midwives (OR: 10.91; 95% CI: 3.18–37.49) and OB/GYNs (OR: 3.64; 95% CI: 1.88–7.02). Moreover, the odds of having recommended influenza immunization were significantly higher for those OB/GYNs and midwives with more positive attitude (OR: 1.27; 95% CI: 1.01–1.62) (Table data in the Supplementary material). The most frequent reasons why HCWs had not recommended vaccination against influenza included the belief it was outside the scope of their practice (46%) and, among those, 91.3% were midwives and 8.7% PCPs; vaccine hesitancy among pregnant people (36%); and lack of knowledge (26%) or time (22%). The strategies perceived as able to improve vaccine uptake during pregnancy, were offering vaccinations during prenatal care visits (58.8%), counseling and advising women about the availability, effectiveness and safety of vaccinations during pregnancy (55.9%), improving accessibility to vaccination services (e.g., flexible schedules, weekend vaccination sessions) (44.3%), making vaccine available at the hospital (38.7%), enhancing the midwives role in vaccinating pregnant people (21.1%) (Table 3).

**Table 2:** Results of the regression model for potential determinants of the outcomes of interest.

Variables	OR	95% CI	*p*
Model 1: Outcome: having advised women about influenza vaccination during pregnancy Log-likelihood = −208.32917; Prob > chi2 < 0.001; Obs = 415			
Knowledge score about vaccinations and VPDs during pregnancy, continuous	1.63	1.26–2.12	<0.001
Attitude score about immunization during pregnancy, continuous	1.20	1.09–1.33	<0.001
Profession			
OB/GYN^*^	1.00		
Midwife	0.30	0.17–0.54	<0.001
PCP	0.83	0.40–1.74	0.629
Number of years in practice, continuous	1.02	1.01–1.05	0.039
Female gender	0.85	0.50–1.44	0.546
Model 2: Outcome: having recommended influenza vaccination during pregnancy Log-likelihood = −216.44751; Prob > chi2 < 0.001; Obs = 415			
Having advised pregnant people about influenza vaccination	5.65	3.35–9.54	<0.001
Attitude score about immunization during pregnancy, continuous	1.16	1.05–1.29	0.005
Profession			
OB/GYN^*^	1.00		
Midwife	0.86	0.46–1.61	0.644
PCP	2.45	1.07–5.59	0.033
Female gender	1.08	0.64–1.80	0.777
Number of years in practice, continuous	1.00	0.98–1.02	0.877
Knowledge score about vaccinations and VPDs during pregnancy, continuous	1.02	0.79–1.32	0.895

^*^Reference category; VPDs, vaccine-preventable diseases; OB/GYN, obstetrician-gynaecologist; PCP, primary care physician.

**Table 3:** Possible strategies to improve vaccine uptake during pregnancy.

Statements (413 respondents)^*^	*N*	%
Offering vaccinations during prenatal care visits	243	58.8
Counseling and advising women about the availability, effectiveness and safety of vaccinations during pregnancy	231	55.9
Improving accessibility to vaccination services	183	44.3
Making vaccines available at the hospital	160	38.7
Enhancing the role of midwives in vaccinating pregnant people	87	21.1
Reminding/offering vaccination through text messages or email	68	16.5

^*^Multiple responses allowed.

### 3.5. Sources of information

Among the sources of information about immunization during pregnancy, HCWs most frequently mentioned conferences/symposiums (85.4%), professional associations (82.6%), and scientific journals (78%). The participants declared to be most satisfied with the information provided by scientific journals (85.1%), followed by professional associations (83.5%) and conferences/symposiums (74.6%). However, three-fifths (60.2%) of the participants wished to receive additional information on the topic.

## 4. Discussion

The National Healthcare Service provides essential assistance relating to pregnancy (e.g., prenatal diagnosis, routine medical visits, etc.), free of charge in public facilities in Italy. HCWs who deliver prenatal care to prospective mothers have a considerable number of opportunities during this period to create vaccination demand by improving awareness. Indeed, despite recommendations, influenza coverage during pregnancy appears less than ideal (34). Given that pregnant people’s hesitancy towards vaccinations remains a public health concern and that vaccine availability coupled with HCWs’ recommendation are the best predictors of vaccination (1), it is critical that HCWs are knowledgeable about the most recent immunization recommendations, and are willing to provide the most up-to-date and relevant information about immunization during pregnancy. With this in mind, the present study sought to understand how HCWs’ knowledge and attitudes towards vaccination might influence the practice of advising and recommending influenza and COVID-19 vaccination during pregnancy.

From the study, three main key points were identified. First and foremost, a sizable portion of the HCWs lacks up-to-date knowledge of the window of time for appropriately administering the influenza vaccine. There is a large body of scientific studies that supports the safety of influenza vaccine in pregnant people during any trimester of pregnancy (35–37). In 2019, the recommendations for the administration of the influenza vaccine have been updated in Italy (38), allowing the vaccine to be safely administered at any stage of pregnancy, in contrast to the earlier recommendations, indicating the vaccine can be given during the 2nd or 3rd trimester only. More than half of the sample stated that only the 2nd or 3rd trimester was the proper time frame for, and this result emphasizes the importance of keeping HCWs up-to-date with the latest evidence-based recommendations. By using evidence-based practices, HCWs are able to deliver the best quality care with the aim of improving patient outcomes by combining their clinical expertise with the best available research evidence. The results of the multiple logistic regression analysis corroborate this, and show that having a high level of knowledge is the primary factor influencing HCWs’ practices to provide pregnant people with information about the influenza vaccination. Subgroup logistic analyses confirmed this finding among midwives and PCPs.

Secondly, more than two thirds of the sample did not recognize that pregnant people are at high risk for severe influenza or COVID-19 complications and one third of the responders considered that the risks of vaccination during pregnancy outweighed the benefits. The tendency to underestimate the risks of contracting a VPD coupled with the overestimation of vaccine side effects, are even more concerning than the low level of knowledge. In fact, positive attitudes were found to be one of the strongest predictor that was positively associated with advising pregnant people to receive the influenza vaccine, according to the results of the regression analyses both in HCWs and in OB/GYNs. Consequently, pregnant people may neglect to take the necessary precautions to reduce their risk of miscarriage or preterm labor as a result of not receiving accurate information from HCWs. It is well known that pregnant people and their unborn children rarely experience serious adverse reactions to vaccinations during pregnancy, and these side effects typically last a short time before disappearing on their own (35, 39, 40). Therefore, it is critical to track what HCWs know and believe about vaccination and how these variables could influence their practices (i.e., informing and recommending immunization). Targeted education and awareness initiatives could be used to combat misconceptions and worries about the safety and effectiveness of vaccines. Tailored evidence-based strategies to improve uptake by engaging HCWs as credible sources of information are strongly needed (41). Moreover, tools to support HCWs in addressing reasons for undervaccination have to be developed and put into action.

Thirdly, a significant portion of HCWs did not fully comply with the expected practices of informing and recommending influenza and COVID-19 vaccinations during pregnancy, which has implications for public health, especially in terms of reducing VPDs and potential severe complications. It is worth noting that the proportion of OB/GYNs recommending influenza vaccination to pregnant people (72.1%) increased compared to a previous Italian study (16.4%) (42) and it is similar to more recent national studies (43, 44). It seems arguable that HCWs need to better understand their responsibilities for educating and counseling pregnant people about the benefits of the influenza vaccine. The latter is also supported by the finding that, among those who did not recommend vaccination the majority of respondents stated that recommending vaccination during pregnancy was outside the scope of their practice, and midwives were the most prevalent professional category in this group. Among the HCWs, midwives represent the first primary healthcare providers for expectant mothers (45). Indeed, throughout her pregnancy, a woman commonly has several interactions with a midwife, who could actively participate in the campaign to increase vaccination uptake when discussing prenatal vaccination. In Italy, however, the midwife’s role as a reliable resource for women’s counseling is largely neglected. As such, the need for adequate training of midwives to ensure proper management of vaccination during pregnancy is essential. Indeed, a recent survey among Italian midwives reported that the odds of informing and recommending COVID-19 vaccination to pregnant people was positively associated to awareness of their role in the prevention of the disease and having received information about the COVID-19 vaccination for pregnant people by official government organizations or scientific journals (46). Allowing midwives to administer vaccines may also increase the vaccination rates for expectant mothers and their families, according to previous studies (47, 48). In light of these data, they should be actively involved in the vaccination process for this to occur. The culture of vaccination among HCWs, should be focused on empowering and enabling pregnant people to make informed decisions. It has been shown that increasing HCWs’ expertise in this area can enhance the outcomes of challenging vaccination conversations with those who exhibit vaccine hesitancy (49).

### 4.1. Limitations

When interpreting the study’s findings, some limitations should be acknowledged. First, as the data were self-reported, they were susceptible to social desirability and recall bias. Nonetheless, social desirability bias was potentially minimized by ensuring the anonymity and confidentiality of the collected data. Second, because it was impossible to examine the characteristics of non-responders, a non-response bias should be considered. A mixed strategy, which has been shown to increase response rate, was utilized to collect the data in order to reduce this probability. Out of 650 HCWs approached, 415 HCWs (63.8%) completed the questionnaire. Moreover, PCPs are less representative than OB/GYNs and midwives, as well as HCWs from Sicily region are less in number than those from Calabria and Campania regions. However, since every HCW standing in the frame of all eligible subjects had an equal chance of being included in the sample, selection bias has been minimized. Third, the data were collected in three Italian regions, which might not represent the whole country. Although we cannot exclude that our results pertain only to these regions, it is reasonable to suppose that the results could be referred to the Southern part of Italy. Finally, because the purpose of this study was descriptive, the relationship between variables and outcomes of interest is more speculative, and readers should be wary of drawing causal conclusions based on the observed differences. However, this was not the primary aim of the study, as we planned to evaluate HCWs’ practices regarding advising and recommending immunization during pregnancy, useful to inform policymakers.

## 5. Conclusion

In order to close the gap between recommendations and implementation, it is pivotal to understand the perspectives of HCWs who have the responsibility to advise and recommend vaccines during pregnancy. The gathered data shed light on such attributes that influence the likelihood that evidence will be applied to improve the impact of preventative interventions. Future research and interventions should be generated to promote adherence to evidence-based practices among HCWs.

## Data availability statement

The dataset presented in this study can be found in online repositories. The name of the repository and accession number can be found in Mendeley Data repository (doi: 10.17632/wg2kmmm3rz.1).

## Ethics statement

The study involving human participants was reviewed and approved by the Calabria Centre Local Human Research Ethics Committee (ID No. 275/2021/07/15). The participants provided their written informed consent to participate in this study.

## Author contributions

FL participated in the conceptualization and in the design of the study, and contributed to the data analysis and interpretation. CPP, GDP, and FL collected the data. EAC and FL contributed to the preparation of the first draft of the manuscript. AB, the principal investigator, designed the study, coordinated and supervised data collection, was responsible for the statistical analysis and interpretation, and wrote the final article. All authors contributed to the article and approved the submitted version.

## Funding

The authors received financial support from Sanofi for the cost of the data collection and the publication fee. However, the funding source had no role in study design, data collection, data analysis, data interpretation, or writing of the paper.

## Conflict of interest

The authors declare that the research was conducted in the absence of any commercial or financial relationships that could be construed as a potential conflict of interest.

## Publisher’s note

All claims expressed in this article are solely those of the authors and do not necessarily represent those of their affiliated organizations, or those of the publisher, the editors and the reviewers. Any product that may be evaluated in this article, or claim that may be made by its manufacturer, is not guaranteed or endorsed by the publisher.

## References

[1] D’Alessandro A Napolitano F D’Ambrosio A Angelillo IF. Vaccination knowledge and acceptability among pregnant women in Italy. Hum Vaccin Immunother. (2018) 14:1573–9. doi: 10.1080/21645515.2018.1483809, PMID: 29863958 PMC6067873

[2] Nunes MC Cutland CL Jones S Downs S Weinberg A Ortiz JR. Efficacy of maternal influenza vaccination against all-cause lower respiratory tract infection hospitalizations in young infants: results from a randomized controlled trial. Clin Infect Dis. (2017) 65:1066–71. doi: 10.1093/cid/cix497, PMID: 28575286 PMC5848298

[3] Zerbo O Modaressi S Chan B Goddard K Lewis N Bok K. No association between influenza vaccination during pregnancy and adverse birth outcomes. Vaccine. (2017) 35:3186–90. doi: 10.1016/j.vaccine.2017.04.074 28483192

[4] World Health Organization. Strategic advisory Group of Experts on immunization. The global vaccine action plan 2011–2020. Review and lessons learned. Geneva: World Health Organization (2019).

[5] Ministero della Salute. Piano nazionale prevenzione vaccinale 2017–2019. Available at: https://www.salute.gov.it/portale/vaccinazioni/dettaglioContenutiVaccinazioni.jsp?lingua=italiano&id=4828&area=vaccinazioni&menu=vuoto (Accessed December 20, 2022).

[6] Omer SB Munoz FM Jamieson DJ. Maternal immunization. Obstet Gynecol. (2017) 133:739–53. doi: 10.1097/AOG.0000000000003161 30913173

[7] World Health Organization. How to implement influenza vaccination of pregnant women. Geneva: World Health Organization (2017).

[8] Agenzia Italiana del Farmaco. Vaccini anti-influenzali per la stagione 2020–2021 registrati secondo la procedura Centralizzata dell’Agenzia Europea dei Medicinali (European Medicines Agency, EMA). (2019) Available at: https://www.aifa.gov.it/documents/20142/101823/vaccini_influenzali:centralizzati_2020-2021.pdf (Accessed February 15, 2023).

[9] Centers for Disease Control and Prevention. Guidelines for vaccinating pregnant women. CdcGov (2012) 60. Available at: http://www.cdc.gov/nip/Publications/preg_guide.htm (Accessed January 12, 2023).

[10] Fabiani M Volpe E Faraone M Bella A Rizzo C Marchetti S. Influenza vaccine uptake in the elderly population: individual and general practitioner’s determinants in Central Italy, Lazio region, 2016–2017 season. Vaccine. (2019) 37:5314–22. doi: 10.1016/j.vaccine.2019.07.054, PMID: 31331778

[11] Kilich E Dada S Francis MR Tazare J Chico RM Paterson P. Factors that influence vaccination decision-making among pregnant women: a systematic review and meta-analysis. PLoS One. (2020) 15:e0234827. doi: 10.1371/journal.pone.0234827, PMID: 32645112 PMC7347125

[12] Kan T Zhang J. Factors influencing seasonal influenza vaccination behaviour among elderly people: a systematic review. Public Health. (2018) 156:67–78. doi: 10.1016/j.puhe.2017.12.007, PMID: 29408191 PMC7111770

[13] Bianco A Della Polla G Angelillo S Pelullo CP Licata F Angelillo IF. Parental COVID-19 vaccine hesitancy: a cross-sectional survey in Italy. Expert Rev Vaccines. (2022) 21:541–7. doi: 10.1080/14760584.2022.2023013, PMID: 34949136

[14] Limaye RJ Opel DJ Dempsey A Ellingson M Spina C Omer SB. Communicating with vaccine-hesitant parents: a narrative review. Acad Pediatr. (2021) 21:S24–9. doi: 10.1016/j.acap.2021.01.018, PMID: 33958087

[15] World Health Organization. Interim recommendations for use of the Moderna mRNA-1273 vaccine against COVID-19. Interim guidance - 25 January 2021. Available at: https://apps.who.int/iris/bitstream/handle/10665/338862/WHO-2019-nCoV-vaccines-SAGE_recommendation-mRNA-1273-2021.1-eng.pdf?sequence=5&isAllowed=y (Accessed April 11, 2023).

[16] World Health Organization. Interim recommendations for use of the Pfizer– BioNTech COVID-19 vaccine, BNT162b2, under emergency use listing. Interim guidance - 8 January 2021. Available at: https://apps.who.int/iris/bitstream/handle/10665/338862/WHO-2019-nCoV-vaccines-SAGE_recommendation-mRNA-1273-2021.1-eng.pdf?sequence=5&isAllowed=y (Accessed April 12, 2023).

[17] Italian Obstetric Surveillance System (ItOSS) Istituto Superiore di Sanità. Indicazioni ad interim su “Vaccinazione contro il COVID - 19 in gravidanza e allattamento” - 09 gennaio 2021. Available at: https://www.iss.it/documents/20126/0/Documento+ItOSS+su+vaccino+ANTI+covid-19+in+gravidanza+e+allattamento_9+gennaio.pdf/5f6c170c-3d37-d6b5-a1e7-b0b728dc1ba6?t=1610194643183 (Accessed April 12, 2023).

[18] World Health Organization. Interim recommendations for use of the Pfizer– BioNTech COVID-19 vaccine, BNT162b2, under emergency use listing. Interim guidance - 19 November 2021. Available at: https://WHO/2019-nCoV/vaccines/SAGE_recommendation/BNT162b2/2021.1 (Accessed April 10, 2023).

[19] World Health Organization. Interim recommendations for use of yhe Moderna mRNA-1273 vaccine against COVID-19. Interim guidance - 19 November 2021. Available at: https://apps.who.int/iris/bitstream/handle/10665/349300/WHO-2019-nCoV-vaccines-SAGE-recommendation-mRNA-1273-2021.3-eng.pdf?sequence=5&isAllowed=y (Accessed April 12, 2023).

[20] Centers for Disease Control and Prevention. COVID-19 vaccines while pregnant or breastfeeding. Available at: https://www.cdc.gov/coronavirus/2019-ncov/vaccines/recommendations/pregnancy.html (Accessed February 16, 2023).

[21] ItOSS - Italian Obstetric Surveillance System, ISS - Istituto Superiore di Sanità. Indicazioni ad interim su “Vaccinazione contro il COVID-19 in gravidanza e allattamento” -Aggiornamento del 7 ottobre 2022. (2022) https://www.epicentro.iss.it/vaccini/pdf/indicazioni-vaccini-covid-gravidanza-allattamento.pdf (Accessed April 11, 2023).

[22] Vilca LM Martínez C Burballa M Campins M. Maternal care providers’ barriers regarding influenza and pertussis vaccination during pregnancy in Catalonia, Spain. Matern Child Health J. (2018) 22:1016–24. doi: 10.1007/s10995-018-2481-6, PMID: 29417364

[23] Loubet P Nguyen C Burnet E Launay O. Influenza vaccination of pregnant women in Paris, France: knowledge, attitudes and practices among midwives. PLoS One. (2019) 14:e0215251. doi: 10.1371/journal.pone.0215251, PMID: 31022211 PMC6483190

[24] Dvalishvili M Mesxishvili D Butsashvili M Kamkamidze G McFarland D Bednarczyk RA. Knowledge, attitudes, and practices of healthcare providers in the country of Georgia regarding influenza vaccinations for pregnant women. Vaccine. (2016) 34:5907–11. doi: 10.1016/j.vaccine.2016.10.033, PMID: 27773474

[25] Dubé E Gagnon D Kaminsky K Green CR Ouakki M Bettinger JA. Vaccination during pregnancy: Canadian maternity care providers’ opinions and practices. Hum Vaccin Immunother. (2020) 16:2789–99. doi: 10.1080/21645515.2020.1735225, PMID: 32271655 PMC7733901

[26] Napolitano F Bianco A D’Alessandro A Papadopoli R Angelillo IF. Healthcare workers’ knowledge, beliefs, and coverage regarding vaccinations in critical care units in Italy. Vaccine. (2019) 37:6900–6. doi: 10.1016/j.vaccine.2019.09.053, PMID: 31564452

[27] Power ML Leddy MA Anderson BL Gall SA Gonik B Schulkin J. Obstetrician-gynecologists’ practices and perceived knowledge regarding immunization. Am J Prev Med. (2009) 37:231–4. doi: 10.1016/j.amepre.2009.05.019, PMID: 19596538

[28] Kissin DM Power ML Kahn EB Williams JL Jamieson DJ MacFarlane K. Attitudes and practices of obstetrician-gynecologists regarding influenza vaccination in pregnancy. Obstet Gynecol. (2011) 118:1074–80. doi: 10.1097/AOG.0b013e3182329681, PMID: 22015875 PMC4608446

[29] Bonville CA Cibula DA Domachowske JB Suryadevara M. Vaccine attitudes and practices among obstetric providers in New York state following the recommendation for pertussis vaccination during pregnancy. Hum Vaccin Immunother. (2015) 11:713–8. doi: 10.1080/21645515.2015.1011999, PMID: 25714987 PMC4525680

[30] Licata F Di Gennaro G Cautela V Nobile CGA Bianco A. Endodontic infections and the extent of antibiotic overprescription among Italian dental practitioners. Antimicrob Agents Chemother. (2021) 65:e0091421–1. doi: 10.1128/AAC.00914-21, PMID: 34252306 PMC8448121

[31] Maertens K Braeckman T Top G van Damme P Leuridan E. Maternal pertussis and influenza immunization coverage and attitude of health care workers towards these recommendations in Flanders, Belgium. Vaccine. (2016) 34:5785–91. doi: 10.1016/j.vaccine.2016.09.055, PMID: 27742214

[32] Mijović H Greyson D Gemmell E Trottier MÈ Vivion M Graham JE. Perinatal health care providers’ approaches to recommending and providing pertussis vaccination in pregnancy: a qualitative study. CMAJ Open. (2020) 8:E377–82. doi: 10.9778/cmajo.20190215, PMID: 32414884 PMC7239636

[33] StataCorp. Stata statistical software: Release 17. College Station, TX: StataCorp LLC (2021).

[34] Ministero della Salute. Piano Nazionale Prevenzione Vaccinale PNPV 2023–2025. https://www.salute.gov.it/imgs/C_17_notizie_5029_0_file.pdf (Accessed February 13, 2023).

[35] Regan AK Munoz FM. Efficacy and safety of influenza vaccination during pregnancy: realizing the potential of maternal influenza immunization. Expert Rev Vaccines. (2021) 20:649–60. doi: 10.1080/14760584.2021.1915138 33832397

[36] McMillan M Porritt K Kralik D Costi L Marshall H. Influenza vaccination during pregnancy: a systematic review of fetal death, spontaneous abortion, and congenital malformation safety outcomes. Vaccine. (2015) 33:2108–17. doi: 10.1016/j.vaccine.2015.02.068, PMID: 25758932

[37] Pasternak B Svanström H Mølgaard-Nielsen D Krause G Emborg H-D Melbye M. Risk of adverse fetal outcomes following. JAMA. (2012) 308:165–74. doi: 10.1001/jama.2012.6131, PMID: 22782418

[38] Ministero Della Salute. Vaccinazioni raccomandate per le donne in età fertile e in gravidanza Aggiornamento novembre. (2019). Available at: http://www.trovanorme.salute.gov.it/norme/renderNormsanPdf?anno=2019&codLeg=71540&parte=1%20&serie=null (Accessed April 27, 2023).

[39] Giles ML Krishnaswamy S Macartney K Cheng A. The safety of inactivated influenza vaccines in pregnancy for birth outcomes: a systematic review. Hum Vaccin Immunother. (2019) 15:687–99. doi: 10.1080/21645515.2018.1540807, PMID: 30380986 PMC6605784

[40] Etti M Calvert A Galiza E Lim S Khalil A Le Doare K. Maternal vaccination: a review of current evidence and recommendations. Am J Obstet Gynecol. (2022) 226:459–74. doi: 10.1016/j.ajog.2021.10.041, PMID: 34774821 PMC8582099

[41] Pelullo CP Della Polla G Napolitano F di Giuseppe G Angelillo IF. Healthcare workers’ knowledge, attitudes, and practices about vaccinations: a cross-sectional study in Italy. Vaccines. (2020) 8:148. doi: 10.3390/vaccines8020148, PMID: 32225018 PMC7348811

[42] Esposito S Tremolati E Bellasio M Chiarelli G Marchisio P Tiso B. Attitudes and knowledge regarding influenza vaccination among hospital health workers caring for women and children. Vaccine. (2007) 25:5283–9. doi: 10.1016/j.vaccine.2007.05.011, PMID: 17580099

[43] Riccò M Vezzosi L Gualerzi G Balzarini F Capozzi VA Volpi L. Knowledge, attitudes, beliefs and practices of obstetrics-gynecologists on seasonal influenza and pertussis immunizations in pregnant women: preliminary results from North-Western Italy. Minerva Ginecol. (2019) 71:288–97. doi: 10.23736/S0026-4784.19.04294-1, PMID: 30938116

[44] Scatigna M Appetiti A Pasanisi M D’Eugenio S Fabiani L Giuliani AR. Experience and attitudes on vaccinations recommended during pregnancy: survey on an Italian sample of women and consultant gynecologists. Hum Vaccin Immunother. (2022) 18:1–8. doi: 10.1080/21645515.2021.1894061, PMID: 33956557 PMC8920149

[45] Homer CSE Javid N Wilton K Bradfield Z. Vaccination in pregnancy: the role of the midwife. Front Glob Womens Health. (2022) 3:929173. doi: 10.3389/fgwh.2022.929173, PMID: 36353468 PMC9637860

[46] Miraglia del Giudice G Della Polla G Folcarelli L Napoli A Punzo R Peracchini M. Midwives’ knowledge, attitudes, and practice regarding COVID-19 vaccination for pregnant women: a nationwide web-based survey in Italy. Vaccines. (2023) 11:222. doi: 10.3390/vaccines11020222, PMID: 36851098 PMC9961665

[47] Massot E Epaulard O. Midwives’ perceptions of vaccines and their role as vaccinators: the emergence of a new immunization corps. Vaccine. (2018) 36:5204–9. doi: 10.1016/j.vaccine.2018.06.050, PMID: 29970300

[48] Frawley JE McKenzie K Cummins A Sinclair L Wardle J Hall H. Midwives’ role in the provision of maternal and childhood immunisation information. Women Birth. (2020) 33:145–52. doi: 10.1016/j.wombi.2019.02.006, PMID: 30853352

[49] Carman R Andrew L Devine A. The knowledge, attitudes and beliefs of midwives on the vaccination coverage rates in Perth’s aboriginal children. BMC Public Health. (2021) 21:1845. doi: 10.1186/s12889-021-11907-1, PMID: 34641835 PMC8507363

