# Supplementary material for: Immunization during pregnancy: do healthcare workers recommend vaccination against influenza?
Source: Front Public Health. 2023 Jun 2;11:1171142. doi: 10.3389/fpubh.2023.1171142 (PMC10272812; doi:10.3389/fpubh.2023.1171142)
Supplement: Supplementary file 1 [file Table_1.DOCX]

| **Table 1. Results of the regression model for potential determinants of the outcomes of interest** **among different professional groups** | | | |
| --- | --- | --- | --- |
| ***Model 1: Outcome:*** *having advised women about influenza vaccination during pregnancy among obstetrician-gynaecologist*  *Log-likelihood = -123.86424; Prob > chi2 < 0.0025; Obs = 269* | | | |
| **Variables** | **OR** | **95% CI** | ***p*** |
| Attitude score about immunization during pregnancy, continuous | 1.25 | 1.09-1.44 | 0.002 |
| Number of years in practice, continuous | 1.04 | 1.00-1.08 | 0.026 |
| Knowledge score about vaccinations and VPDs during pregnancy, continuous | 1.29 | 0.89-1.86 | 0.177 |
| Female gender | 0.87 | 0.45-1.65 | 0.660 |
| ***Model 2: Outcome:*** *having advised women about influenza vaccination during pregnancy among primary care physicians*  *Log-likelihood =-26.306241; Prob > chi2 < 0.0142 ; Obs = 56* | | | |
| **Variables** | **OR** | **95% CI** | ***p*** |
| Knowledge score about vaccinations and VPDs during pregnancy, continuous | 3.71 | 1.37-10.02 | 0.010 |
| Attitude score about immunization during pregnancy, continuous | 1.18 | 0.90-1.54 | 0.225 |
| Number of years in practice, continuous | 0.97 | 0.92-1.03 | 0.383 |
| Female gender | 0.55 | 0.14-2.19 | 0.398 |
| ***Model 3: Outcome:*** *having advised women about influenza vaccination during pregnancy among midwives*  *Log-likelihood = -52.222978; Prob > chi2 < 0.0007; Obs = 90* | | | |
| **Variables** | **OR** | **95% CI** | ***p*** |
| Knowledge score about vaccinations and VPDs during pregnancy, continuous | 2.05 | 1.28-3.27 | 0.003 |
| Attitude score about immunization during pregnancy, continuous | 1.20 | 0.97-1.48 | 0.093 |
| Number of years in practice, continuous | 1.02 | 0.99-1.07 | 0.215 |
| Female gender | 1.77 | 0.42-7.38 | 0.435 |
| ***Model 4: Outcome:*** *having recommended influenza vaccination during pregnancy among obstetrician-gynaecologist*  *Log-likelihood = -144.50341; Prob > chi2 < 0.0001; Obs = 269* | | | |
| **Variables** | **OR** | **95% CI** | ***p*** |
| Having advised pregnant people about influenza vaccination | 3.64 | 1.88-7.02 | <0.001 |
| Number of years in practice, continuous | 1.04 | 1.00-1.07 | 0.024 |
| Attitude score about immunization during pregnancy, continuous | 1.16 | 1.01-1.36 | 0.037 |
| Female gender | 1.31 | 0.73-2.34 | 0.363 |
| Knowledge score about vaccinations and VPDs during pregnancy, continuous | 0.92 | 0.65-1.30 | 0.643 |
| ***Model 5: Outcome:*** *having recommended influenza vaccination during pregnancy among primary care physicians*  *Log-likelihood = -15.691923; Prob > chi2 < 0.0002; Obs = 56* | | | |
| **Variables** | **OR** | **95% CI** | ***p*** |
| Having advised pregnant people about influenza vaccination | 21.47 | 2.91-158.39 | 0.003 |
| Attitude score about immunization during pregnancy, continuous | 1.27 | 0.86-1.87 | 0.231 |
| Number of years in practice, continuous | 0.95 | 0.87-1.04 | 0.269 |
| Female gender | 0.39 | 0.06-2.66 | 0.333 |
| Knowledge score about vaccinations and VPDs during pregnancy, continuous | 1.41 | 0.40-4.98 | 0.594 |
| ***Model 6: Outcome:*** *having recommended influenza vaccination during pregnancy among midwives*  *Log-likelihood = -44.288307; Prob > chi2 < 0.0001; Obs = 90* | | | |
| **Variables** | **OR** | **95% CI** | ***p*** |
| Having advised pregnant people about influenza vaccination | 10.91 | 3.18-37.49 | <0.001 |
| Number of years in practice, continuous | 0.94 | 0.90-0.99 | 0.018 |
| Attitude score about immunization during pregnancy, continuous | 1.27 | 1.00-1.62 | 0.048 |
| Female gender | 2.50 | 0.43-14.64 | 0.308 |
| Knowledge score about vaccinations and VPDs during pregnancy, continuous | 1.12 | 0.70-1.79 | 0.639 |
| *Reference category; VPDs: vaccine-preventable diseases; OB/GYN: obstetrician-gynaecologist; PCP: primary care physician | | | |
